# Therapeutic Targeting of mTOR in T-Cell Acute Lymphoblastic Leukemia: An Update

**DOI:** 10.3390/ijms19071878

**Published:** 2018-06-26

**Authors:** Camilla Evangelisti, Francesca Chiarini, James A. McCubrey, Alberto M. Martelli

**Affiliations:** 1 CNR Istituto di Genetica Molecolare, Unità di Bologna, 40136 Bologna, Italy; camilla.evangelisti@cnr.it (C.E.); francesca.chiarini@cnr.it (F.C.); 2 Istituto Ortopedico Rizzoli, 40136 Bologna, Italy; 3 Department of Microbiology & Immunology, Brody School of Medicine, East Carolina University, Greenville, NC 27834, USA; 4 Department of Biomedical and Neuromotor Sciences, University of Bologna, 40126 Bologna, Italy

**Keywords:** mTOR, T-cell acute lymphoblastic leukemia, targeted therapy, combination therapy

## Abstract

T-cell acute lymphoblastic leukemia (T-ALL) is an aggressive blood malignancy that arises from the clonal expansion of transformed T-cell precursors. Although T-ALL prognosis has significantly improved due to the development of intensive chemotherapeutic protocols, primary drug-resistant and relapsed patients still display a dismal outcome. In addition, lifelong irreversible late effects from conventional therapy are a growing problem for leukemia survivors. Therefore, novel targeted therapies are required to improve the prognosis of high-risk patients. The mechanistic target of rapamycin (mTOR) is the kinase subunit of two structurally and functionally distinct multiprotein complexes, which are referred to as mTOR complex 1 (mTORC1) and mTORC2. These two complexes regulate a variety of physiological cellular processes including protein, lipid, and nucleotide synthesis, as well as autophagy in response to external cues. However, mTOR activity is frequently deregulated in cancer, where it plays a key oncogenetic role driving tumor cell proliferation, survival, metabolic transformation, and metastatic potential. Promising preclinical studies using mTOR inhibitors have demonstrated efficacy in many human cancer types, including T-ALL. Here, we highlight our current knowledge of mTOR signaling and inhibitors in T-ALL, with an emphasis on emerging evidence of the superior efficacy of combinations consisting of mTOR inhibitors and either traditional or targeted therapeutics.

## 1. Introduction

T-cell acute lymphoblastic leukemia (T-ALL) is an aggressive disease that represents 10–15% of ALL cases in children and up to 25% in adults [1,2]. T-ALL is a genetically heterogeneous disorder caused by the accumulation of molecular alterations acting in a multistep pathogenic process [3]. While ≥80% of pediatric patients with T-ALL can expect to be cured [4], among adults younger than 60 years treated with conventional chemotherapy, the survival rates are in the range of 40–50%, and older patients have a much worse prognosis [5]. Although the use of high-dose multiagent chemotherapy results in a survival advantage, many patients still relapse and eventually experience refractory leukemia, which is associated with a poor likelihood of survival [5]. For example, only 20% of relapsed pediatric patients can be cured with current salvage protocols [6]. Moreover, especially childhood T-ALL survivors are at increased risk of developing long-term adverse health outcomes, including secondary malignancies due to the use of genotoxic drugs [7]. Therefore, novel, more effective and less toxic treatments are desired to improve the outcome of T-ALL patients, as well as their quality of life both during and after therapy.

Our increased knowledge of genetic alterations has significantly contributed to identify oncogenetic drivers and signaling cascades regulating T-ALL pathophysiology. This has opened the possibility of targeting pathways that are critical to prevent and/or treat relapse [8]. The mechanistic target of rapamycin (mTOR) is a key effector of signaling networks that are aberrantly regulated in T-ALL and negatively affect patient outcome [9]. In this context, it is also important to emphasize that very recent evidence has demonstrated that high mTOR expression is an independent negative prognosticator of clinical outcome to induction chemotherapy in T-ALL patients [10].

Here, we summarize and discuss recent advances in understanding and targeting mTOR in T-ALL settings with the aim of highlighting possible less toxic therapeutic strategies for improving the outcome of chemoresistant/refractory patients.

## 2. mTOR and Its Complexes: Structure, Activation, and Functions

**Figure 1:**
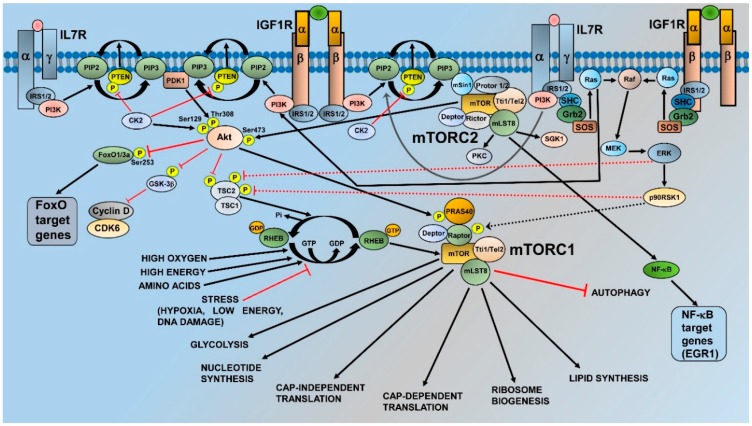
Regulation and functions of mechanistic target of rapamycin complex 1 (mTORC1) and mTORC2. For details, see the text. Black arrows indicate stimulatory events, while red lines indicate inhibitory events.

mTOR is a 289-kDa protein encoded in humans by the *MTOR* gene mapping to chromosomal band 1p36.2 [11]. mTOR is an evolutionary conserved member of the phosphatidylinositol 3-kinase (PI3K)-related kinase (PIKK) family of protein kinases [12], and acts as the catalytic subunit of two large multiprotein complexes, which are referred to as mTOR complex 1 (mTORC1) and mTORC2. These complexes share some components, which include Tel2-interacting protein 1 (Tti1)/Tel2 complex, Dishevelled, Egl-10 and Pleckstrin (DEP) domain-containing mTOR-interacting protein (Deptor), and mammalian lethal with SEC13 protein 8 (mLST8) [13]. mTORC1 is defined by the association of mTOR with the regulatory-associated protein of mTOR (Raptor), which is a protein that is fundamental for mTORC1 assembly, stability, regulation, and substrate specificity [14]. Moreover, mTORC1 comprises proline-rich Akt substrate 1 40 kDa (PRAS40), which blocks mTORC1 activity until growth factor receptor signaling unlocks PRAS40-mediated mTORC1 inhibition [15]. The activation of mTORC1 is achieved by growth factors, cytokines, hormones, amino acids, high energy levels, and oxygen through multiple mechanisms. In contrast, intracellular and environmental stresses (low ATP levels, hypoxia, DNA damage) are powerful repressors of mTORC1 activity [13] (Figure 1). For the scope of this article, it is important to emphasize that growth factors, such as insulin-like growth factor-1 (IGF-1) or cytokines [interleukin (IL) 7, for example] activate PI3K. PI3K generates at the plasma membrane phosphatidylinositol 3,4,5 trisphosphate (PIP3) from phosphatidylinositol 4,5 bisphosphate (PIP2). PIP3 recruits to the plasma membrane phosphoinositide-dependent kinase 1 (PDK1) and Akt that is phosphorylated by PDK1 at Thr308 [16]. Akt phosphorylates tuberous sclerosis complex 2 (TSC2) at Thr1462 [17]. TSC2 is a GTPase activating protein (GAP) that functions in association with TSC1 to lock the small G-protein, RAS homolog enriched in brain (Rheb) in a GDP-bound, inactive state. Akt-mediated TSC1/TSC2 complex inhibition consequently allows Rheb to accumulate in a GTP-bound state, whereby Rheb-GTP binds and activates mTORC1 [18]. Moreover, Akt phosphorylates the mTORC1 inhibitor PRAS40 at Thr246. This phosphorylation causes PRAS40 dissociation from Raptor, allowing mTORC1 activation [19]. Also, the rat sarcoma (RAS)/rapidly accelerated fibrosarcoma (Raf)/mitogen-activated protein kinase (MEK)/extracellular signal-regulated kinase (ERK)/p90 ribosomal S6 kinase 1 (p90RSK1) cascade impinges on mTORC1, as both ERK and p90RSK1 phosphorylate TSC2 (at Ser664 and Ser1798, respectively), thereby inhibiting the TSC1/TSC2 complex and triggering Rheb-dependent mTORC1 activation [20]. Moreover, p90RSK1 can phosphorylate Raptor, causing mTORC1 activation [21]. As to the functions of mTORC1, they include the upregulation of cap-dependent and cap-independent translation, increased glycolysis, enhanced lipid and nucleotide synthesis, as well as positive regulation of ribosome biogenesis through the RNA polymerase (Pol) I-dependent and Pol III-dependent transcription of the different classes of ribosomal RNAs [13,22,23]. In contrast, mTORC1 is a repressor of autophagy [24] (Figure 1).

mTORC2 is characterized by the interactions of mTOR with the rapamycin independent companion of mTOR (Rictor), mammalian stress-activated protein kinase interacting protein 1 (mSin1), and protein observed with rictor (Protor) 1 or 2 [13]. Rictor is necessary for mTORC2 assembly, stability, and substrate interactions [25], while mSin1 is a repressor of mTORC2 kinase activity [26]. Nevertheless, it also drives mTORC2 localization to the plasma membrane, where Sin1-mediated mTORC2 inhibition is relieved in response to the growth factor receptor-dependent activation of PI3K [27]. Regarding Protor1, it may be involved in enabling mTORC2 to phosphorylate serum and glucocorticoid-activated kinase 1 (SGK1) [28]. In contrast to mTORC1, our knowledge of the control of mTORC2 activity is limited. However, recent evidence has highlighted that plasma membrane localization is a critical aspect of mTORC2 regulation. Indeed, the pleckstrin homology (PH) domain of mSin1 interacts with the mTOR kinase domain to restrain mTOR activity. PIP3, which is synthesized by PI3K at the cell membrane, binds mSin1-PH to release its inhibition on mTOR, thereby triggering mTORC2 activation [27].

As for the roles of mTORC2, this complex phosphorylates several members of the AGC family of protein kinases [29]. These include protein kinase C (PKC) isoforms α/γ/δ/ε/ζ and SGK1 [13] (Figure 1). However, the most important and best known function of mTORC2 is the phosphorylation of Akt at Ser473, which fully activates the kinase activity of Akt [30]. Ser473 phosphorylation is required for Akt-mediated phosphorylation of Forkhead box O1/3a (FoxO1/3a) trascription factors, but not for that of other Akt targets, such as TSC2 and glycogen synthase kinase3β (GSK3β) [26]. In light of its substrates, mTORC2 is mainly involved in the control of cytoskeletal remodeling, cell migration, proliferation, and survival [13]. Nevertheless, it has been recently demonstrated that mTORC2 is a repressor of chaperone-mediated autophagy [31]. Furthermore, mTORC2 increases lipid synthesis, whereby promoting carcinogenesis [32].

## 3. Activation of mTORC1 and mTORC2 in T-ALL Cells

We will now review the multiple mechanisms that explain why the activities of both mTORC1 and mTORC2 are aberrantly regulated in T-ALL.

### 3.1. Phosphatase and Tensin Deleted on Chromsome 10

Compelling evidence indicates that phosphatase and tensin deleted on chromosome 10 (PTEN), which is the main negative regulator of the PI3K/Akt/mTOR cascade [33,34,35], plays a key role in the activation of this pathway in T-ALL cells [36]. PTEN dephosphorylates PIP3, thus yielding PIP2 and blunting PI3K activity (Figure 1). However, the gene encoding for PTEN is frequently either deleted or mutated in human T-ALL cell lines and primary samples [37], resulting in PI3K/Akt/mTOR upregulation. Interestingly, a very recent retrospective study has demonstrated that PTEN mutations, when combined with additional genetic anomalies (NOTCH1, FBXW7, and RAS mutations) and a high white blood cell count, were associated with a higher risk of relapse in childhood T-ALL [38].

Moreover, even when expressed in its wild-type form, PTEN is phosphorylated at a cluster of residues (Ser380/Thr382/Ser385) in the C-terminal, resulting in the downregulation of PTEN lipid phosphatase activity and high PIP3 levels [39]. Casein kinase 2 (CK2), which is overexpressed in T-ALL [40], has been identified as the kinase responsible for PTEN phosphorylation and inactivation in leukemic cells [41]. Furthermore, CK2 phosphorylates Akt at Ser129 (Figure 1). This phosphorylation positively contributes to Akt activity and increases Akt association with the chaperone protein heat shock protein 90 (HSP90), thus protecting Akt from protein phosphatase 2A (PP2A) activity at Thr308 [42]. However, PTEN is also a target of neurogenic locus notch homolog protein 1 (NOTCH1) signaling, as we will see later on in this article.

Apart from controlling PIP3 levels, PTEN induces miR-26b expression by regulating the differential expression of the Ikaros transcription factor isoforms that are upstream of miR-26b [43]. Accordingly, the levels of miR-26b were lower in PTEN-deficient mouse and human T-ALL cells. Intriguingly, it was shown that miR-26b negatively controls the expression of PI3K p110δ, which is a PI3K catalytic subunit important for PIP3 generation in T-ALL [43,44,45]. The overexpression of miR-26b decreased Ser473 p-Akt levels (which is indicative of mTORC2 inhibition), while either shRNA to PI3K p110δ or a PI3K p110δ-selective inhibitor (CAL-101) reduced the viability of T-ALL cell lines [43]. Overall, these findings highlighted a novel mechanism through which PTEN deficiency could result in a further increase in PI3K/Akt/mTOR signaling independently from PTEN lipid phosphatase activity.

### 3.2. NOTCH1 Signaling

NOTCH1 is a key oncogenetic driver of T-ALL, and NOTCH1 activating mutations occur in ≥50% of T-ALL patients [46]. Hairy and enhancer of split-1 (HES1) transcription factor, which is downstream of NOTCH1, represses PTEN expression and contributes to enhancing PI3K/AKT/mTOR signaling in NOTCH1-dependent T-ALL [47]. Furthermore, the NOTCH1/HES1 axis is somehow responsible for decreased PP2A activity on Thr308 and Ser473 p-Akt, resulting in the activation of downstream effectors, including mTORC1 [48].

Additional NOTCH1-dependent mechanisms that contribute to decreased PTEN levels have been identified. PTEN can be targeted and downregulated by miR-19 [49] or c-Myelocytomatosis oncogene protein (c-Myc) [50]. Moreover, NOTCH1 could control mTORC1 signaling through yet another mechanism, as documented by a study in which the treatment of T-ALL cell lines with a γ-secretase inhibitor (GSI) targeting NOTCH1 resulted in the dephosphorylation of mTORC1 downstream targets, including eukaryotic translation initiation factor 4E-binding protein 1 (4E-BP1), p70 S6 ribosomal protein kinase 1 (p70S6K1), and S6 ribosomal protein (S6RP), independently of PI3K/Akt activity. These effects on mTORC1 could be rescued by expression of the intracellular domain of NOTCH1 (ICN1) and mimicked by dominant negative mastermind-like transcriptional coactivator 1 (MAML1), which is a NOTCH1 regulator [51]. Furthermore, the expression of c-Myc opposed GSI-induced mTORC1 inhibition, thus implicating c-Myc as an intermediary between NOTCH1 and mTORC1 [51]. This observation could be related to c-Myc being a transcriptional repressor of TSC2; hence, high levels of c-Myc could result in upregulated mTORC1 activity, independently from PI3K/Akt [52].

Besides mTORC1, NOTCH1 has been proposed to somehow regulate mTORC2 activity as well, at least in a murine model of T-ALL, where hematopoietic bone marrow precursors were transduced to express ICN1 and transplanted into recipient mice [53]. Animals that received cells with Rictor conditional knockout showed at most a modest decrease in bone marrow and circulating leukemic cells. However, the median survival of these animals almost doubled when donor marrow was programmed to delete Rictor; moreover, the mice displayed decreased organ (lung, kidney, liver) invasion by ICN1-driven leukemic cells. Intriguingly, the expression of Nuclear factor κ-light-chain-enhancer of activated B cells (NF-κB) target genes (Bcl2a1, Nfkb2, and CCR7) was significantly decreased in the Rictor-depleted circulating T-ALL cells, whereas selected FoxO1/3 target genes (Il7ra, Sell, and S1p1) were not [53]. In this context, it is important to emphasize that C-C chemokine receptor type 7 (CCR7) has been shown to act as an important determinant of NOTCH1-driven T-ALL pathogenesis and death because of its critical role in regulating the trafficking of the leukemic cells into tissues [54]. Therefore, this study provided evidence that mTORC2 is an important determinant of the capacity of active NOTCH1 to induce NF-κB activity and CCR7 expression (most likely through Akt phosphorylation at Ser473 [55]), as well as accelerated tissue invasion and death in a murine T-ALL model. Nevertheless, a different group demonstrated that, in a NOTCH1-driven murine model of T-ALL, Rictor deletion—hence, mTORC2 inactivation—affected the activity of FoxO transcription factors as well. Indeed, in mice where Rictor expression was genetically suppressed, leukemia progression was hampered by a slower cell proliferation and decreased infiltration of organs such as the lung, liver, and kidney. This was accompanied by decreased phosphorylation of Ser473 p-Akt and Ser253 p-FoxO3a, as well as by increased expression of FoxO3a target genes (Figure 1), including those encoding for negative regulators of cell cycle progression, such as p21^Cip1^ and p27^Kip1^. In contrast, the expression levels of positive regulators of cell cycle, cyclin-dependent kinase (CDK) 1 and 4, were decreased in Rictor-deleted T-ALL cells [56]. Moreover, this study documented that the absence of Rictor led to the overexpression of chemotaxis-related proteins, such as CCR2, CCR4, and C-X-C chemokine receptor (CXCR) 4, which most likely contributed to increased migration and the homing of Rictor-deficient T-ALL cells to the spleen, whereas migration to bone marrow was negatively affected. However, FoxO3a downregulation by shRNA did not affect the migration of T-ALL cells, suggesting a different type of control [56], although previous studies had documented that FoxO transcription factors are somehow involved in CXCR4 expression [57].

Overall, the results by Lee et al. [53] and Hua et al. [56] support the concept that in murine NOTCH1-mutated T-ALL models, mTORC2 is a critical regulator of leukemia progression that impacts a variety of genes targeted by both NFκB and FoxO3a.

Deptor has been identified as an mTORC1/mTORC2 component that is under the control of NOTCH1, as NOTCH1 directly binds to and activates Deptor promoter in T-ALL cells [58]. Deptor depletion by shRNA abolished cell proliferation, attenuated glycolytic metabolism, and enhanced cell death, whereas ectopically expressed Deptor significantly promoted cell growth and glycolysis. Furthermore, Deptor ablation delayed T-ALL onset in a xenograft model. These effects were mostly related to the control of Akt phosphorylation at both Thr308 and Ser473, as Deptor depletion inhibited Akt activation, while its overexpression enhanced it [56]. These findings may appear surprising at a first glance, as Deptor inhibits both mTORC1 and mTORC2 [59]. However, it was found that while Deptor depletion increased p70S6K1 phosphorylation, its overexpression inhibited p70S6K1 phosphorylation [56]. This suggested that Deptor activates Akt at least in part through the inhibition of mTORC1 activity, as reported in several studies (e.g., [60]).

### 3.3. RAS Signaling

RAS proteins include Harvey-RAS (H-RAS), neuroblastoma-RAS (N-RAS), and Kirsten-RAS (K-RAS) [61]. They are a family of small GTPases acting as molecular switches that oscillate between an inactive GDP-bound and an active GTP-bound status. RAS genes are the most frequently mutated genes in human cancer [62]. RAS proteins transduce signals from a variety of cell receptors, including receptor tyrosine kinases (RTKs) and cytokine receptors, to downstream effectors such as PI3K/Akt and MEK/ERK (Figure 1). By doing so, they regulate a plethora of functions that are fundamental for both healthy and tumor cells [63]. Activating RAS mutations have the potential for inducing T-ALL in murine models when combined with other genetic anomalies, including enhancer of Zeste 2 (EZH2) inactivation [64] as well as NOTCH1 [65,66] or IL7 receptor (IL7R) α chain mutations [67]. RAS signaling is overactive in about ≥50% of childhood T-ALL patients [68]. N-RAS and K-RAS activating mutations seem to occur more frequently in early T-cell progenitor (ETP)-ALL [69] than in other subtypes [70,71,72]. Of note, ETP-ALL is a T-ALL subtype characterized by a poor outcome [73]. In this context, it is important to highlight that in a murine model of T-ALL evoked by K-RAS activation, Raptor deficiency dramatically inhibited the cell cycle progression of T-cell progenitors and prevented leukemia development, thus emphasizing the key role played by mTORC1 in this setting [74].

However, RAS signaling cascade upregulation could also arise from mutations or alterations in the activity/expression of key regulatory components of the RAS pathway, including RAS guanine nucleotide-releasing protein 1 (RASGRP1, that is frequently overexpressed in human T-ALL cell lines and primary samples) [75,76], or RAS GTPase-activating proteins (RAS-GAPs), such as neurofibromin 1 or p120 RAS-GAP [77,78]. In murine T-ALL cells with increased RASGRP1 expression, RASGRP1 contributed to cytokine receptor-activated RAS pathway that stimulated the proliferation of T-ALL cells in vivo [75]. Remarkably, RASGRP1 overexpression in T-ALL cells seems to impinge primarily on PI3K/Akt rather than on MEK/ERK signaling [79,80].

### 3.4. RTK Signaling

Aberrant signals originating from RTKs have been implicated in PI3K/Akt/mTOR upregulation in T-ALL. A well-documented example is increased IGF1/IGF1 receptor (IGF1R) activity [81]. Indeed, IGF1R levels are increased both transcriptionally [82,83] and post-transcriptionally [84] by NOTCH1 in T-ALL cells [46]. As to the source of IGF1, a recent study has revealed how, in the thymic microenvironment of murine T-ALL models and T-ALL primary patient samples, leukemic cells overexpressed IGF1R while tumor-associated dendritic cells (DCs) synthesized and released IGF1, which drove T-ALL growth ex-vivo [85]. Importantly, it has been shown that IGF1/IGF1R signaling contributes to proliferation and survival not only of the bulk T-ALL cells, but also of cells endowed with leukemia-initiating activity [82].

Another RTK that is overexpressed and cooperates with PTEN deficiency to activate PI3K/Akt/mTOR in T-ALL cell lines and primary samples is neurotrophic tyrosine receptor kinase type 2 (NTRK2, also known as TrkB) [44]. Interestingly, the NTRK2 transcript levels were consistently higher in PTEN-deficient T-ALL cell lines and primary samples compared with PTEN wild-type cells. However, the significance of such an inverse correlation is still unclear.

### 3.5. IL7 Signaling

IL7/IL7R signaling has been documented to play a critical role in mTOR activation in T-ALL. IL7 and IL7R are essential for normal T-cell development and homeostasis, whereas disregulated IL7/IL7R activity promotes T-ALL [8]. In T-ALL, gain of function mutations of IL7Rα, which could be detected in about 10% of pediatric patients, resulted in the activation of PI3K/Akt/mTOR signaling [86,87]. Interestingly, both IGF1/IGF1R and IL7/IL7R activate not only PI3K/Akt but also the MEK/ERK module [81,88] (Figure 1). However, at least in human T-ALL cell lines, PI3K/Akt signaling was dominant over MEK/ERK in mediating cell proliferation and/or survival [81], although in the article by Triplett et al. [85], MEK/ERK activation by IGF1/IGF1R was detected in T-ALL cells co-cultured with thymic DCs. Moreover, IGF1/IGF1R and IL7/IL7R displayed non-overlapping roles in the control of T-ALL cell line growth [81]. However, further studies will be required to determine to which extent these findings apply to primary patient samples. In any case, it should be emphasized that NOTCH1 is a transcriptional activator also of the gene encoding IL7Rα [89].

### 3.6. Integrins and Chemokines

Integrin and chemokine signals are known for activating both PI3K/Akt and MEK/ERK [90,91]; hence, they have the potential for positively impacting mTORC1 and mTORC2. Accordingly, integrins and chemokines are important for regulating several aspects of T-ALL cell biology, including proliferation, survival, drug-resistance, migration, and infiltration of the central nervous system [2,8,92,93].

### 3.7. PI3K Activating Mutations

Although genetic anomalies of the PI3K p110α catalytic subunit are frequently detected in some types of solid cancers [94,95], they seem to be exceedingly rare in T-ALL [96].

### 3.8. mTOR Mutations

An emerging theme in mTOR biology is the identification of activating mutations that could confer increased sensitivity to mTOR inhibitors. Several such mutations have been identified in solid cancer cell lines and patients [97]. However, at present, the only activating mutation described in T-ALL cells is C1483Y, which has been identified in the MOLT-16 human cell line [98]. This mutation occurs in the FRAP, ATM, TRRAP (FAT) domain of mTOR, and leads to lower levels of Raptor bound to mTORC1/mTORC2 and higher levels of Rictor interacting with mTORC2. Therefore, the final effect is an increase in the activity of both mTORC1 and mTORC2 [99]. However, MOLT-16 cells are PTEN-deleted [100]. Therefore, their PTEN status also most likely contributes to mTORC1/mTORC2 activation.

## 4. Roles of mTORC1 and mTORC2 in T-ALL

The roles of the individual mTOR complexes were recently explored by shRNA knockdown strategy in a mouse model where T-ALL had been induced by ΔTrkA, which is a mutant of TrkA isolated from a patient with acute myeloid leukemia [101]. Some of the T-ALL clones also displayed PTEN mutations, abrogating the lipid phosphatase activity, and NOTCH1-activating mutations. While ΔTrkA was sufficient for upregulating mTORC1 (most likely through MEK/ERK signaling, as Akt was barely active in this model), increased mTORC2 activity required both inactivating PTEN and activating NOTCH1 mutations. Separate depletion of either Raptor (mTORC1) or Rictor (mTORC2) reduced the proliferation rate and the size of T-ALL cells, but was not sufficient to induce apoptosis [102]. In contrast, knockdown of eukaryotic translation initiation factor 4E (eIF4E, the rate limiting factor of mTORC1-dependent mRNA-translation [103]) had a dramatic impact, leading to significantly reduced cell size and proliferation, as well as remarkable apoptosis. Similar results were obtained using 4EGI-1, which is a small molecule that abrogates cap-dependent translation through direct binding to eIF4E [104]. As expected, either eIF4E knockdown or treatment with 4EGI-1 reduced the expression of key oncogenetic proteins and shifted the mitochondrial outer membrane toward an apoptosis-facilitating state [102]. At first glance, these findings are difficult to reconcile with Raptor knockdown by shRNA in T-ALL cells being ≥96%; therefore, cap-dependent translation should have been almost completely switched off. However, it should be considered that MEK/ERK/p90RSK1 signaling directly converges on eIF4E through mitogen-activated protein kinase interacting kinases (MNKs), partially bypassing mTORC1 [105]. Since MER/ERK was constitutively active in the murine T-ALL model used by Schwarzer et al. [102], it might be that Raptor knockdown did not attain a level of cap-dependent translation inhibition that was sufficient for inducing apoptosis, whereas eIF4E downregulation was more effective in this respect.

## 5. Therapeutic Targeting of mTORC1 and mTORC2 in T-ALL Cells: Preclinical Studies

mTOR was originally discovered as the target of rapamycin, which is a macrolide antibiotic isolated in 1972 from the bacterium Streptomyces hygroscopicus in the soil collected on Easter Island (Rapa Nui in the local language) [97,106]. Three classes of mTOR inhibitors are at present available: allosteric inhibitors (rapamycin and its derivatives or rapalogs, i.e., RAD001/everolimus, CCI-779/temsirolimus) that mainly target mTORC1 [107]; ATP-competitive dual PI3K/mTOR inhibitors that target PI3K, mTORC1 and mTORC2 [108]; and ATP-competitive mTOR kinase inhibitors (TORKIs) that target mTORC1 and mTORC2, but not PI3K [109].

### 5.1. Allosteric mTOR Inhibitors

Allosteric mTOR inhibitors have proven their efficacy against T-ALL cells in several preclinical studies. This class of drugs mainly exerts cytostatic effects [109]. Accordingly, rapamycin or temsirolimus blocked IL7-dependent T-ALL proliferation and cell cycle progression. mTOR inhibition was accompanied by an increased expression of the CDK inhibitor, p27^Kip1^ [110,111]. Rapamycin or temsirolimus also induced apoptosis of T-ALL cells cultured in the presence of IL7. Apoptotic cell death was characterized by the activation of p53, as documented by upregulated levels of Ser46 p-p53 [111], which is in agreement with previous findings [112]. Moreover, it has been demonstrated that rapamycin restored the expression of other cell cycle negative regulators, p14 and p15, in the MOLT-4 human T-ALL cell line [113]. Interestingly, these effects were related to the demethylation of the promoters of the genes encoding for p14 and p15, as rapamycin decreased total DNA methyltransferase (DNMT) activity in MOLT-4 cells. Although other groups have reported that mTORC1 controls the expression of DNMT1 [114], the molecular mechanism underlying this regulation remains unexplained.

The proapoptotic effects of rapamycin and rapalogs could be significantly increased by co-treatment with drugs that are currently employed in T-ALL patients, including doxorubicin [111,115], idarubicin [116], cyclophosphamide [117], and methotrexate [118]. Moreover, mTOR allosteric inhibitors synergize with glucocorticoids (GCs), that are widely used in current protocols for treating T-ALL [111,119,120]. It should be emphasized here that pediatric T-ALL patients often display GC resistance [121]. These patients, who are classified as prednisone poor responders (PPP), have worse outcome than other T-ALL patients receiving a high-risk adapted therapy [122]. Therefore, GC-resistance represents an important challenge for improving the prognosis of PPP.

In particular, it has been shown that rapamycin downregulated the expression of myeloid leukemia cell differentiation 1 (MCL-1), which is a critical regulator of GC-induced apoptosis, as it sequesters the BH3-only proapoptotic protein B-cell lymphoma-2 (Bcl-2) -like protein 11 (BIM) in GC-resistant CEM T-ALL cells [123]. Wei et al. [123] could not detect an increase in the expression levels of either BIM or p53-upregulated modulator of apoptosis (PUMA), which is another BH3-only proapoptotic protein. However, in a subsequent study that also took advantage of GC-resistant CEM cells, it was reported that rapamycin, when combined with GCs, upregulated GC receptor α isoform as well as BIM [124].

Allosteric mTOR inhibitors also have proven their efficacy in T-ALL cells when combined with other targeted therapeutics, which included inhibitors of NOTCH1 [125], MEK [111], Janus kinase 3 (Jak3) [111], Bcl-2 [126], and glycolysis [127].

More recently, it has been shown that a combination consisting of LEE011 (ribociclib), which is an investigator-grade CDK 4/6 inhibitor, and everolimus, was synergistic in vitro in reducing cell proliferation and increasing the apoptosis of human T-ALL cell lines [128]. The rationale for using this drug combination is that both CDK6 and its upstream regulator, cyclin D3, are frequently upregulated in T-ALL [129,130,131,132]. Cyclin D3 is a downstream target of NOTCH1 signaling in T-ALL [133]. However, CDK4/6 inhibition in cancer cells is usually cytostatic; therefore, monotherapy is unlikely to be optimal [134]. Moreover, CDK4/6 inhibitor could unleash adaptive responses that lead to an acquired resistance to this class of drugs. Some of these responses are orchestrated by mTORC1 or mTORC2 [135]. Indeed, previous studies that have been carried out in solid cancers have demonstrated the efficay of combining a CDK4/6 inhibitor with everolimus [136]. Pikman et al. [128] also investigated the effects of the LEE011 and everolimus combination in an orthotopic mouse model of T-ALL, where MOLT-16 cells were injected into NOD-SCID IL2Rγ^null^ (NSG) mice. They found that the drug combination resulted in a significantly prolonged mice survival compared with either drug alone.

### 5.2. Dual PI3K/mTOR Inhibitors

This class of drugs was originally developed to overcome some of the drawbacks of allosteric mTOR inhibitors, such as the only partial inhibition of mTORC1-dependent translation and the feedback activation of oncogenetic pathways, including PI3K/Akt [97,108,137]. When used in T-ALL cell models, some of these drugs (PI-103, NVP-BEZ235) displayed a more potent proapoptotic activity than rapamycin, and inhibited one of the rapamycin-resistant outputs of mTORC1, i.e., 4E-BP1 phosphorylation [138,139,140]. However, it has been demonstrated that PI-103 upregulated NOTCH1/c-Myc signaling in NOTCH1-mutated T-ALL cell lines, thus leading to an impaired cytotoxic response [141]. Drug combinations consisting of PI-103 and either a GSI or a small molecule c-Myc inhibitor (10058-F4) overcame resistance to the dual PI3K/mTOR targeting agent. Also, dual PI3K/mTOR inhibitors synergized with chemotherapeutic drugs used for T-ALL treatment [139,142]. In particular, NVP-BEZ235 enhanced GC-induced anti-leukemic activity in vitro (cell lines and primary samples) and systemic in vivo models of T-ALL, including a patient-derived xenograft [86,143]. Through the inhibition of Akt, NVP-BEZ235 alleviated the Akt-mediated suppression of GC-induced apoptotic pathways, thus leading to the increased expression of proapoptotic BIM. Furthermore, downregulation of MCL-1 protein by NVP-BEZ235 further contributed to the modulation of GC-resistance by increasing the amount of BIM available to induce apoptosis, especially in PTEN-null T-ALL cells, where the inhibition of Akt only partially overcame Akt-induced BIM suppression [143]. A recently described, highly synergistic drug combination comprises NVP-BEZ235 and calcineurin (Cn) inhibitors, and was effective both in vitro and in vivo in xenograft models [144]. Cn is a Ca^2+^-activated protein phosphatase that plays several key roles in healthy T-cell physiology [145]. However, Cn is also somehow involved in some critical aspects of T-ALL pathophysiology, including GC resistance [121], migration [146], and adhesion [147].

### 5.3. TORKIs

This class of ATP-competitive molecules, which block only the mTOR catalytic domain, was developed to reduce toxicity due to the use of dual PI3K/mTOR inhibitors [97]. TORKIs, when compared with rapamycin, completely blocked in vitro and in vivo the phosphorylation of Ser473 p-Akt and Thr37/46 p-4E-BP1, markedly inhibited cell proliferation, and negatively affected cap-dependent translation under conditions where rapamycin had no effects [148,149]. We investigated the efficacy of two TORKIs, PP-242 and OSI-027, in T-ALL primary samples and cell lines. At variance with rapamycin, we found that the TORKIs induced a marked inhibition of mRNA translation, which led to lower levels of oncogenetic proteins, including MCL-1, survivin, and CDK-2. The inhibitors strongly synergized with both vincristine and the Bcl-2 inhibitor, ABT-263 [150]. Similar results were reported by another group that used the TORKI Torin-2 in human T-ALL cell lines and ICN1-transduced mouse T-ALL cells [151]. Interestingly, Torin-2 increased the expression levels of proapototic genes such as Bcl2l11 (which encodes for BIM) and Bbc3 (encoding for PUMA [152]) as well as of the p53 target gene, Cdkn1b (which encodes for the cell cycle progression, inhibitor p27^Kip1^ [153]). However, it should not be forgotten that p27^Kip1^ expression could be also under the control of FoxO3a [56], which is a target of mTORC2 through Akt (Figure 1). These mechanisms have been confirmed in human NOTCH1 mutated T-ALL Jurkat cells, where treatment with OSI-027, by inhibiting mTORC1-mediated 4E-BP1 phosphorylation, led to the decreased expression of c-Myc and subsequent upregulation of PUMA [154]. In contrast, the inhibition of mTORC2 activity resulted in NFκB–mediated expression of the early growth response 1 (EGR1) gene (Figure 1), which encodes a transcription factor that binds and transactivates the BCL2L11 locus, encoding BIM. Importantly, both of these pathways contributed to T-ALL cell death, which was observed in reponse to OSI-027 treatment [154].

## 6. Clinical Trials

At present, we know the results of three clinical trials where either everolimus or temsirolimus was combined with chemotherapy for treating relapsed/refractory T-ALL patients. The first trial was a Phase I/II study where everolimus was combined with Hyper-CVAD (cyclophosphamide, vincristine, adriamycin, dexamethasone) high-intensity chemotherapy [155] in adult patients with either B-lineage or T-lineage acute leukemia [156]. A partial or complete response was noted in five of 10 heavily pretreated T-ALL patients (median of four prior salvage regimens). Everolimus significantly inhibited the phosphorylation of S6RP, but this did not correlate with clinical response. However, no significant decrease in p-4E-BP1 and p-Akt levels was noted. Interestingly, the combined Hyper-CVAD and everolimus regimen did not result in significantly increased toxicity compared with Hyper-CVAD alone. Therefore, it was concluded that this drug combination was well tolerated and moderately effective in relapsed T-ALL patients [156].

The second study was a Phase I trial of temsirolimus in combination with UKALL R3 re-induction chemotherapy, which was conducted in children and adolescents with second or greater relapse of ALL [157]. Unfortunately, in this study, only one of 16 enrolled patients had T-ALL, while the others had B-ALL. Although the regimen induced remission in seven of 15 evaluable patients, the addition of temsirolimus to reinduction chemotherapy resulted in excessive toxicity and was not tolerable. In any case, the single T-ALL patient did not respond to treatment [157].

The third study was a Phase I trial of everolimus in combination with multiagent chemotherapy (vincristine, prednisone, pegylated asparaginase and doxorubicin) in pediatric ALL patients experiencing a first bone marrow relapse [158]. A total of 22 patients were enrolled, and 19 of them (86%) achieved a second complete remission. Remarkably, everolimus combined with four-drug reinduction chemotherapy was generally well tolerated. However, also in this study, there was only one T-ALL patient who did not respond at all to therapy.

Therefore, given the extremely limited number of T-ALL cases that were enrolled in the aforementioned studies, it is impossible to draw at present any firm conclusions, although it would seem that rapalogs have some potential in combination therapy in adult patients.

Further trials are being performed with rapalogs or the TORKI TAK-228 (Sapanisertib^@^) in combination with a variety of chemotherapeutics (see for example www.clinicaltrials.gov: NCT01614197, NCT03328104, NCT02484430). A very important aspect for development of the field will be the identification of chemotherapeutics that best combine with targeted inhibitors, and whether changes to the schedule and/or dose may alleviate adverse effects.

## 7. Conclusions

The evidence reviewed here demonstrates that mTORC1/mTORC2-generated signals play key roles in the control of T-ALL cell proliferation, survival, metabolism, and drug-resistance, making these complexes critical targets for novel anti-leukemic therapies. Although the use of mTOR inhibitors is continuously yielding a flood of promising preclinical data, initial clinical trials based on these drugs have not resulted in widespread and durable patient responses. As a consequence of these trials, only everolimus and temsirolimus have been approved as anticancer agents in the United States and Europe. They are used for treating advanced renal cell carcinoma, hormone receptor-positive/HER2-negative breast cancer in postmenopausal women, pancreatic and other selected neuroendocrine tumors, adult renal angiomyolipoma associated with TSC disease, pediatric or adult subependymal giant cell astrocytoma with TSC, and relapsed/refractory mantle cell lymphoma [97].

The limited effectiveness of mTOR targeting was initially explained on the basis of the activation of compensatory signaling pathways unleashed by mTOR inhibitors in cancer cells [97]. However, more recently, it became apparent that the low efficacy of these drugs could also depend on other reasons; these include, but are not limited to, the emergence of inhibitor treatment-resistant mTOR mutations [159], intratumor signaling network heterogeneity [80,160] due to the uneven clonal evolution of cancer [137,161] or an acidic tumor microenvironment [162].

Preclinical data strongly indicate that identifying combinations, either with targeted agents or with chemotherapy, might be the key to unleashing the full potential of mTOR inhibitors in T-ALL patients, as we have highlighted in this review. Early clinical data support this claim in other cancer types [163,164,165], although it will have to be conclusively documented that better responses are not accompanied by unacceptable toxicities [166]. Allosteric mTOR inhibitors have been tested in a limited number of clinical trials for treating relapsed/refractory ALL patients in combination with polychemotherapy. These trials have revealed that this class of drugs was also quite well tolerated in childhood patients in general, except for one study. Nevertheless, well-known adverse effects of everolimus and temsirolimus include hyperglycemia, dyslipidemia, mouth ulceration, stomatitis, increased susceptibility to infections, interstitial pneumonitis, vomiting, and diarrhea [97,167,168,169].

The depth and duration of target inhibition as well as the safety profiles of these inhibitors might be improved through the use of intermittent dosing schedules, which could lead to a better drug exposure with more effective target inhibition and fewer adverse effects, as seen in other cancer types [170,171,172].

A key issue in the field of targeted therapy is the identification of biomarkers that could predict accurately inhibitor efficacy. Regarding the field of mTOR inhibitors, our knowledge is virtually non-existent, as potential biomarkers that were identified in preclinical studies unfortunately have not been subsequently validated in clinical trials [173]. Techniques such as kinase activity profiling [174,175], computational analysis [176], and next-generation sequencing [177] should provide a deeper insight into active signal transduction networks and point out critical signaling hubs, new potential druggable targets, as well as drug-sensitive and drug-resistant T-ALL patients. However, it is likely that an integrated approach comprising drug sensitivity, proteomic, phosphoproteomic, and genotypic analyses of primary leukemic cells could be the key for identifying the determinants of sensitivity to targeted compounds [178].

A better understanding of the effects of targeted inhibitors on the immunosuppressive leukemic microenvironment could improve therapeutic approaches [179]. Indeed, recent evidence pointed out that B-ALL cells induced the inhibition of Akt/mTORC1 signaling and glucose metabolism that drove T-cell functional impairment, while an enforced Akt/mTORC1 signaling rescued T-cell metabolism and partially improved anti-leukemia immunity [180]. Therefore, the use of mTOR inhibitors could further blunt immunological responses against leukemic cells. However, another recent report demonstrated that an Akt inhibitor counteracted Th17 cell-induced resistance to daunorubicin in a preclinical model of B-ALL [181].

Targeted therapy is one of the mainstays of personalized cancer medicine, which also includes companion diagnostic [182,183,184]. However, the implementation of targeted agents in T-ALL therapy remains a difficult challenge due to a wide variety of disease-specific and patient-specific factors, such as the co-existence of multiple driver mutations, interconnected signal transduction pathways, age, comorbidities, psychosocial health, and socio-economic status [182]. For example, aberrant NOTCH1 signaling is considered a very promising target for the innovative treatment of T-ALL patients [185]. However, there is a paucity of published studies regarding the clinical use of NOTCH1 inhibitors in T-ALL [186,187], and the same holds true for mTOR inhibitors.

Nevertheless, the field of anti-tumor mTORC1/mTORC2-targeted therapies has progressed rapidly over the past 10 years. We are confident that, as our knowledge of mTOR biology continuously evolves, so too will our capacity to refine these novel treatments for ameliorating T-ALL patient outcomes.

## Abbreviations

4E-BP1	Eukaryotic translation initiation factor 4E-binding protein 1
Bcl-2	B-cell lymphoma-2
BIM	Bcl-2-like protein 11
CCR	C-C chemokine receptor
CDK	Cyclin dependent kinase
CK2	Casein kinase 2
c-Myc	c-Myelocytomatosis oncogene protein
Cn	Calcineurin
CVAD	Cyclophosphamide, vincristine, adriamycin, dexamethasone
CXCR	C-X-C chemokine receptor
DCs	Dendritic cells
DEP	Dishevelled, Egl-10 and Pleckstrin
Deptor	DEP domain-containing mTOR-interacting protein
DNMT	DNA methyltransferase
EGR1	Early Growth Response 1
eIF4E	Eukaryotic translation initiation factor 4E
ERK	Extracellular signal-regulated kinase
ETP	Early T-cell-progenitor
EZH2	Enhancer of Zeste 2
FAT	FRAP, ATM, TRRAP
FoxO1/3a	Forkhead box O1/3a
GAP	GTP-ase activing protein
GCs	Glucocorticoids
GSI	γ-secretase inhibitor
Grb2	Growth factor receptor-bound protein 2
GSK3β	Glycogen synthase kinase 3β
H-RAS	Harvey-RAS
HER2	human epidermal growth factor receptor 2
HES1	Hairy and enhancer of split-1
HSP90	Heat shock protein 90
JAK3	Janus kinase 3
ICN1	Intracellular polypeptide of NOTCH1
IGF-1	Insulin-like growth factor-1
IGF1R	Insulin-like growth factor-1 receptor
IL	Interleukin
IL7R	Interleukin 7 receptor
IRS1/2	Insulin receptor substrate 1/2
K-RAS	Kirsten-RAS
MAML1	Mastermind like transcriptional coactivator 1
MCL-1	Myeloid leukemia cell differentiation
MEK	Mitogen-activated protein kinase kinase
mLST8	Mammalian lethal with SEC13 protein 8
MNKs	Mitogen-activated protein kinase interacting kinases
mSin1	Mammalian Stress-activated protein kinase interacting protein 1
mTOR	Mechanistic target of rapamycin
mTORC1	mTOR complex 1
N-RAS	Neuroblastoma-RAS
NF-κb	Nuclear factor κ-light-chain-enhancer of activated B cells
NOTCH1	Neurogenic locus notch homolog protein 1
NSG	NOD-SCID IL2Rγ^null^
NTRK2	Neurotrophic tyrosine receptor kinase
p70S6K1	p70 Ribosomal protein S6 kinase 1
p90RSK1	p90 ribosomal S6 kinase 1
PDK1	Phosphoinositide-dependent kinase 1
PH	Pleckstrin homology
PI3K	Phosphatidylinositol 3-kinase
PIKK	PI3K-related kinase
PIP2	Phosphatidylinositol 4,5 bisphosphate
PIP3	Phosphatidylinositol 3,4,5 trisphosphate
PKC	Protein kinase C
Pol	RNA polymerase
PP2A	Protein phosphatase 2A
PPP	Prednisone poor responders
PRAS40	Proline-rich Akt substrate 1 40
Protor	Protein observed with Rictor
PTEN	Phosphatase and tensin deleted on chromosome 10
PUMA	p53 upregulated modulator of apoptosis
Raf	Rapidly accelerated fibrosarcoma
Raptor	Regulatory associated protein of mTOR
RAS	Rat sarcoma
RAS-GAP	RAS GTPase activating protein
RASGRP1	RAS guanine nucleotide-releasing protein 1
Rb	Retinoblastoma protein
Rheb	RAS homolog enriched in brain
Rictor	Rapamycin independent companion of tor
RTK	Receptor tyrosine kinase
S6RP	S6 ribosomal protein
SGK1	Serum and glucocorticoid-activated kinase 1
SOS	Son of sevenless homolog
T-ALL	T-cell acute lymphoblastic leukemia
TORKIs	ATP-competitive mTOR kinase inhibitors
TSC1	Tuberous scelerosis complex 1
TSC2	Tuberous sclerosis complex 2
Tti1	Tel2-interacting protein 1
